# A Sprouty4 Mutation Identified in Kallmann Syndrome Increases the Inhibitory Potency of the Protein towards FGF and Connected Processes

**DOI:** 10.3390/ijms22042145

**Published:** 2021-02-21

**Authors:** Astrid Stütz, Anna Z. M. Kamptner, Hedwig Sutterlüty

**Affiliations:** Institute of Cancer Research, Department of Medicine I, Comprehensive Cancer Center, Medical University of Vienna, A-1090 Vienna, Austria; astrid.stuetz@gmx.at (A.S.); anna.kamptner@gmx.net (A.Z.M.K.)

**Keywords:** Spry4, Sprouty protein, Kallmann syndrome, FGFR1, FGF-mediated signaling, MAPK pathway

## Abstract

Kallmann syndrome is the result of innate genetic defects in the fibroblast growth factor (FGF) regulated signaling network causing diminished signal transduction. One of the rare mutations associated with the syndrome alters the Sprouty (Spry)4 protein by converting the serine at position 241 into a tyrosine. In this study, we characterize the tyrosine Spry4 mutant protein in the primary human embryonic lung fibroblasts WI-38 and osteosarcoma-derived cell line U2OS. As demonstrated in a cell signaling assay, Spry4 gains the capability of inhibiting FGF, but not epithelial growth factor (EGF)-induced signaling as a consequence of the tyrosine substitution. Additionally, migration of normal embryonic lung fibroblasts and osteosarcoma-derived cells is potently inhibited by the tyrosine Spry4 variant, while an effect of the wildtype Spry4 protein is hardly measureable. Concerning cell proliferation, the unaltered Spry4 protein is ineffective to influence the WI-38 cells, while the mutated Spry4 protein decelerates the cell doubling. In summary, these data emphasize that like the other mutations associated with Kallmann syndrome the described Spry4 mutation creates a hyperactive version of a selective inhibitory molecule and can thereby contribute to a weakened FGF signaling. Additionally, the study pinpoints a Spry4 variation expanding the applicability of Spry4 in a potential cancer therapy.

## 1. Introduction

Kallmann syndrome belongs to a group of clinical syndromes summarized as congenital hypogonadotropic hypogonadism and is characterized by incomplete pubertal development accompanied with anosmia or hyposmia. In the absence of any hypothalamic-pituitary organic development, the extreme pubertal delay or arrest is caused by a defect in synthesis, secretion, or action of gonadotropin releasing hormone associated with a migratory arrest of the dependent neurons. With a lower penetrance other phenotypes including skeletal abnormalities, dental and renal agenesis, midline malformations, and hearing loss are observed [1].

At the molecular level Kallmann syndrome and hypogonadotropic hypogonadism is linked to disrupted fibroblast growth factor (FGF) signaling. The most frequently implicated gene is the X-chromosomal-linked KAL1 gene encoding for anosmin-1. This protein enhances FGF signaling by direct physical interactions with the fibroblast growth factor receptor (FGFR)-FGF-heparan sulfate proteoglycan (HSPG) complex on the cell surface. The autosomal dominant forms are often the result of loss of function mutations within one of the FGFR1 alleles. Recently, rare mutations in other genes with a function in regulation of FGF signaling have also been discovered. This includes FGF8 and Sprouty (Spry)4 [2,3,4,5]. The Spry4 mutation most frequently found in diseased persons converts a cytosine at position 722 of the coding sequence to an adenine resulting in a protein converting the serine at position 241 to a tyrosine [2,5].

Spry proteins were originally identified as antagonist of a FGFR in drosophila [6]. Subsequent studies revealed that the Spry family members represent modulators of receptor tyrosine kinase (RTK)-driven signaling pathways in higher animals including mammalians. In humans, four homologues fulfill important functions in many signal transduction cascades [7]. Although the specific mode of their action is not fully elucidated, the available data demonstrate an exclusive interference with mitogen activated protein kinase/extracellular-signal regulated kinase (MAPK/ERK) activation especially in response to FGFs as the main function of the Spry proteins [8,9,10]. Nonetheless, occasionally the action of Spry proteins is visible as an influence in signal transmission within the PI3K pathway [11] as well as downstream of phospholipase C [12]. Knockout studies revealed that in mice lack of the single Spry proteins caused phenotypes comparable to the ones observed when growth factors are overrepresented [13,14,15,16] reinforcing the original observation that the protein counteract RTKs. In case of Spry4 knockout, it was reported that the mice are viable and fertile, although a fraction died shortly after birth due to mandible defects. Additionally, growth retardation and polysyndactyly were frequently observed. In the isolated mouse embryonic fibroblasts, ERK activation was intensified in response to FGF, but not to epithelial growth factor (EGF). In combination with the lack of Spry2, the double knockout mice were embryonic lethal [16]. As a negative regulator of signal transduction Spry4 protein is able to exert a tumor-suppressive role as observed for cancer derived from lung [17], breast tissue cells [18], or brain glia [19]. Nonetheless, in contrast to Spry2, Spry4 fails to interfere with the malignant phenotypes of osteosarcoma [20] and ovarian cancer [21]. 

The present study aimed to analyze the functional consequences of the C722A mutation in the coding sequence of Spry4. Using human embryonic lung fibroblasts, we investigated how an altered protein is influencing FGF-induced signal transduction via the MAPK pathway and how cellular processes like migration and cell proliferation are affected by this change.

## 2. Results

### 2.1. A Mutation Associated with Kallmann Disease Capacitates Spry4 as Inhibitor of FGF-Induced Signaling in Normal Human Fibroblasts

In order to investigate the role of the Spry4 mutation associated with Kallmann syndrome, we introduced a cytosine to adenine transversion on position 722 of the Spry4 coding sequence. In the mutated protein the serine at position 241 is now converted to a tyrosine. Since the phenotype characteristics of Kallmann syndrome suggest that an associated causative change of a sequence would result in reduced FGF-mediated signaling and Spry4 is a well-known inhibitor of the downstream MAPK pathway, we first compared mutated Spry^Y241^ with the wildtype Spry4^S241^ concerning their influence on this pathway.

**Figure 1:**
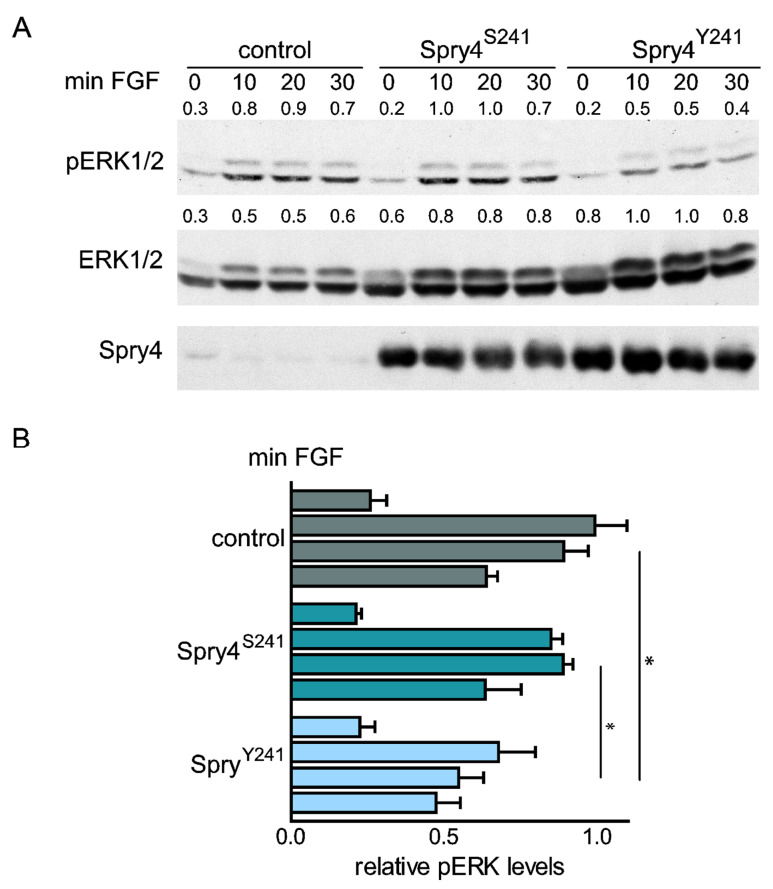
Influence of Spry4 protein variant on FGF2-induced extracellular-signal regulated kinase (ERK)1/2 activation. Human primary lung fibroblasts WI-38 were serum-deprived for 24 h and infected with adenoviruses expressing a control protein, wildtype Spry4(Spry4^S241^) or the mutated Spry4 protein variant (Spry4^Y241^). After an incubation time of 2 days, 10 ng/mL FGF2 were added for 0, 10, 20, and 30 min as indicated. (**A**) Representative Western blots incubated consecutively with antibodies recognizing pERK1/2, total ERK 1/2 and Spry4 are shown. Detected pERK1/2 as well as ERK1/2 bands were quantified using ImageQuant 5.0 as indicated. (**B**) Values for pERK1/2 were normalized to the corresponding values obtained for ERK1/2 expression, arbitrarily setting the highest values as 1. Calculated mean values ± SEM of pERK/ERK ratios from four independent experiments are summarized. By using a one-way ANOVA test in GraphPad Prism, significance was determined. * *p* < 0.05.

Therefore, a cell signaling assay was performed. Serum-starved primary WI-38 cells were infected with viruses expressing the Spry4 variants or a control protein and ERK phosphorylation was determined at different time points after mitogen addition. In serum-deprived WI-38 cells, the expression of both Spry4 variants had no influence on the basal levels of phosphorylated ERK (pERK). In response to FGF2 addition, ERK1/2 proteins were phosphorylated immediately, and pERK levels remained high the first 20 min before they slightly decreased after 30 min (Figure 1A). Expression of the wildtype Spry4^S241^ had no significant influence on the activation pattern of ERK, although in some assays the decline at 30 min was more pronounced (Figure 1). In case of ectopic Spry4^Y241^ expression, the basal pERK levels in arrested cells were unaffected, and like in control cells addition of FGF2 caused an immediate phosphorylation of ERK1/2. However, while in cells expressing a control protein or wildtype Spry4^S241^ the increase of ERK phosphorylation was on average about sixfold, the presence of Spry4^Y241^ diminished the extent of this phosphorylation to a ~4-fold induction. At the time point 20 min after FGF2 addition, the pERK levels were significant lower than in the two reference groups. Additionally, we observed that infection of the cells with the same amount of adenoviruses expressing Spry4^Y241^ or the Spry4 wildtype protein resulted in a comparable yield of Spry4 protein levels. This observation excludes that the variation in position 241 is mainly affecting the half-life of the protein and points towards a functional difference of the two Spry4 variants. 

Therefore, these data clearly support the hypothesis that the conversion of serine 241 to a tyrosine is creating a Spry4 variant which is more effectively inhibiting FGF-induced MAPK activation.

### 2.2. Irrespective of the Variations at Position 241, Spry4 Is Unable to Influence EGF-Mediated ERK Phosphorylation in Normal Embryonic Lung Fibroblasts

**Figure 2:**
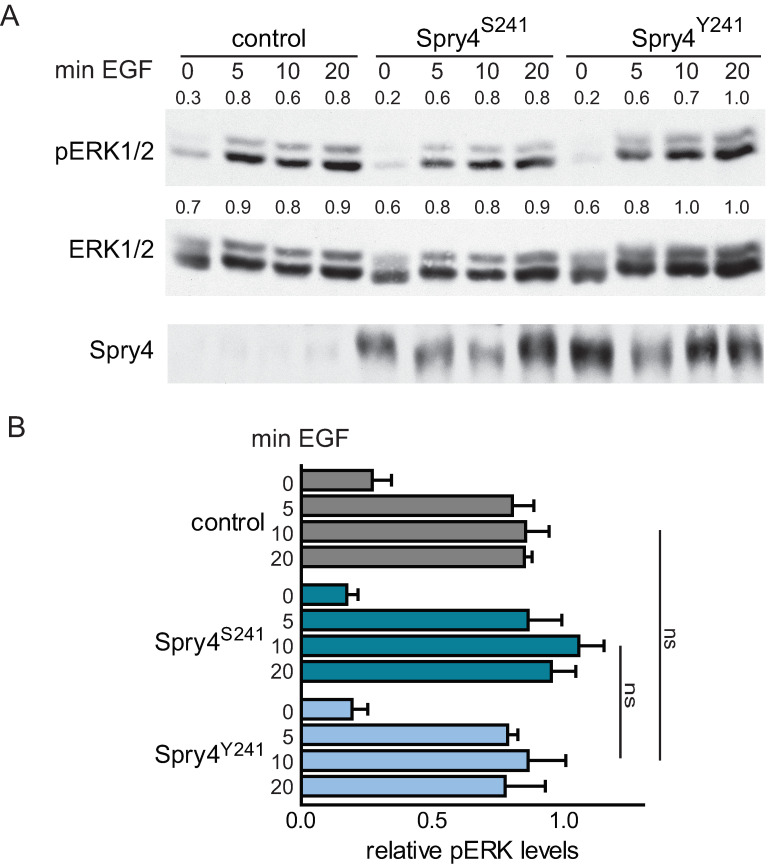
Epithelial growth factor (EGF)-mediated mitogen activated protein kinase (MAPK) activation in the presence of Spry4^S241^ and Spry4^Y241^ in WI-38. A cell signaling assay using 10 ng EGF was performed with serum starved cells overexpressing the indicated proteins. (**A**) Representative immunoblots of one experiment are shown. Antibodies recognizing ERK1/2, pERK1/2 and Spry4 were used to detect the respective proteins. The indicated numbers ahead of the blots were obtained by the densitometric quantification with ImageQuant 5.0 if the highest values were set as 1. (**B**) The graph summarizes three experiments after the bands of pERK and ERK in response to serum were quantified using ImageQuant 5.0, and the highest values of each experiment were set as 1. Significance was determined by a one-way ANOVA test using GraphPad prism software. ns = *p* > 0.5.

To investigate if the mutation of serine 241 to tyrosine is also critical for the cellular response to EGF, we next performed a cell signaling assay using EGF as mitogen. After EGF supplementation, MAPK was induced within 5 min and stayed fully active during the examined time interval. Expression of either Spry4 proteins failed to influence the pERK profile mediated by EGF (Figure 2).

These observations show that the mutation of Spry4 identified in Kallmann syndrome is not affecting the inhibitory potency of the protein on EGF-mediated MAPK activation, suggesting that the effect might be FGF specific.

### 2.3. A Tyrosine at Position 241 Is Augmenting the Capability of Spry4 to Influence the Migratory Potential of WI-38

**Figure 3:**
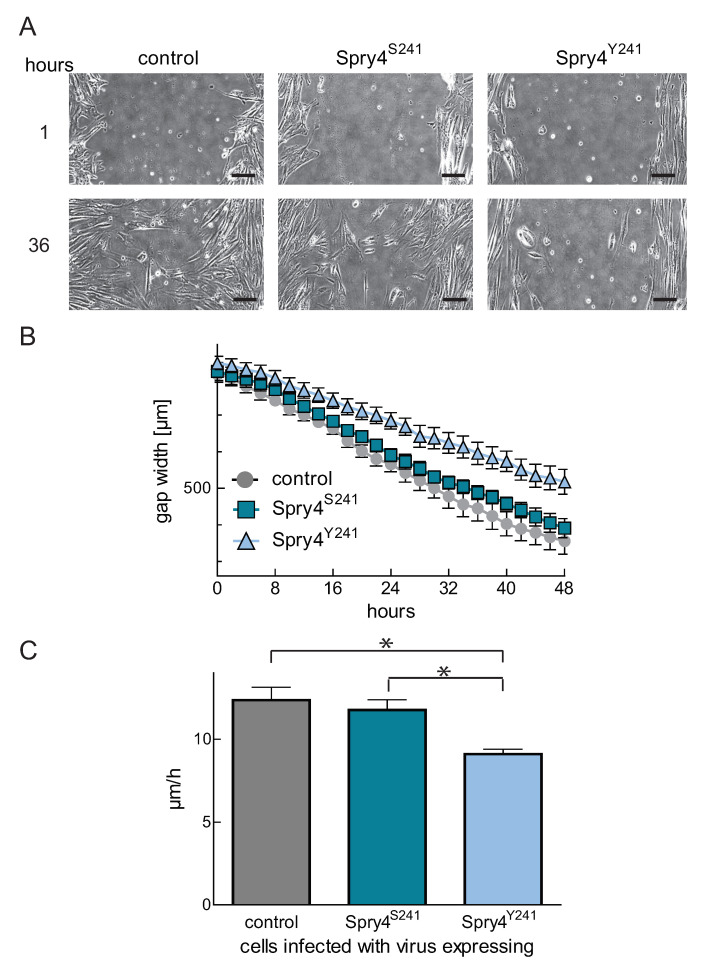
Influence of Spry4 variant expression on cell migration. (**A**) Microscopic pictures with 10× magnification showing gap closure within a period of 36 h. WI-38 cells infected with adenoviruses expressing the indicated proteins. Pictures were taken every hour using a VISITRON Live Cell Imaging System. Scale bar in the right corner of the pictures shows a distance of overall 100 μm. (**B**) Representative curves of distance coverage were obtained by measuring gap widths of three replicative scratches every two hours using ImageJ. (**C**) Using linear regression over the linear range, migration velocities were calculated. Means of at least three experiments ± SEM are summarized as column bar. An unpaired *t*-test was used to acquire significance. * *p* < 0.05.

Cell migration is an important RTK-mediated process contributing to the phenotype observed in patients affected by the Kallmann syndrome. To further verify our hypothesis that the mutation at position 241 creates a hyperactive version of Spry4, we wanted to investigate if the expression of Spry4^S241^ and Spry4^Y241^ proteins modulate the closure of the gap in a Scratch assay. As depicted in Figure 3A, after 36 h scratches with a comparable width were almost closed in control treated and wildtype Spry4^S241^ expressing cells, while in the presence of Spry4^Y241^ the scratch was only slightly closed. A quantitative measurement of the gap width every two hours reveals that Spry4^S241^ restored the cell lawn with a comparable velocity as the control treated cells, while expression of the mutated Spry4 variant was decelerating this effect (Figure 3B). While Spry4 in its wildtype form affects cell migration (11.77 ± 0.685 as compared to 12.36 ± 0.781 µm/h) insignificantly, the mutated version slows down the cells to 9.106 ± 0.305 µm/h (Figure 3C).

These results show that the conversion to tyrosine at position 241 confers a migration-inhibitory potential to Spry4 protein, which can otherwise not be observed in WI-38 cells.

### 2.4. In Primary Embryonic WI-38 Cells, Only the Mutated Spry4 Version Has an Effect on Cell Proliferation

**Figure 4:**
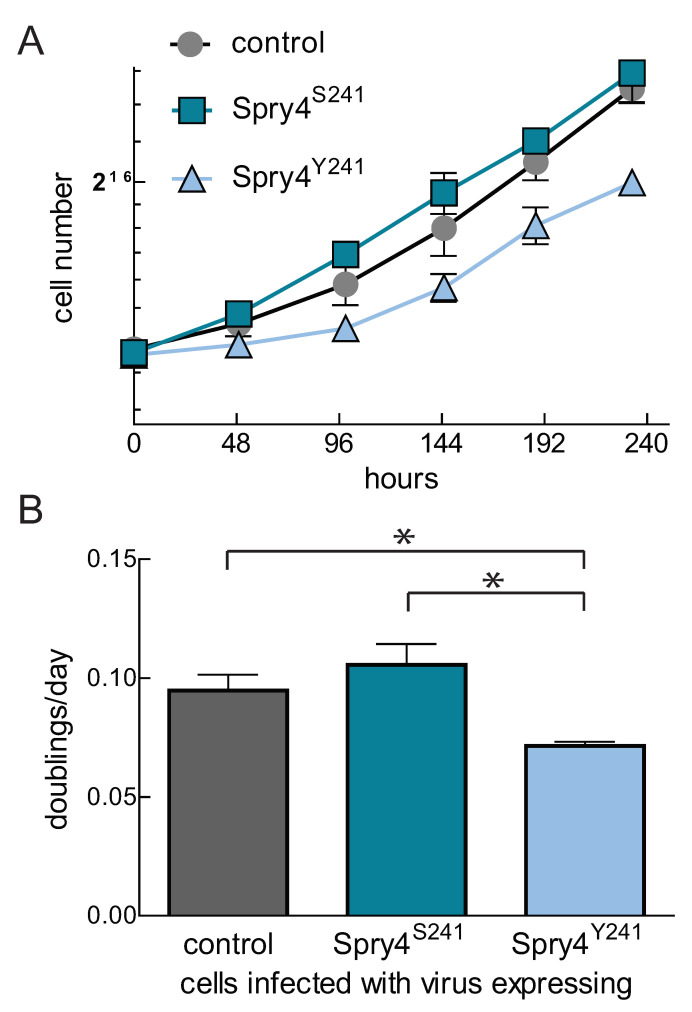
Growth curve analysis of WI-38 with ectopic Spry4^S241^ and Spry4^Y241^ expression. (**A**) The cell number of WI-38 was determined every 48 h over a time period of 10 days. Using a semi-logarithmical scale, cell counts were depicted as growth curve. The presented growth curve summarizes the values of three independent experiments. (**B**) Doubling times of four growth curves of WI-38 were calculated using GraphPad Prism software and presented as mean doublings per day ± SEM. Evaluation of significance was done utilizing one-way analysis of variance (ANOVA). * *p* < 0.05.

To investigate if additionally other RTK-mediated processes are influenced by the mutated Spry4 version, we next analyzed how cell proliferation is affected by the expression of Spry4 protein variants. Therefore, cells were infected with adenoviruses expressing either a control protein, Spry4^S241^ or Spry4^Y241^ before growth curve analyses were performed. As depicted in Figure 4, expression of the wildtype Spry4^S241^ had no inhibitory influence on the proliferation capacity of WI-38 cells. Like control treated primary embryonic human lung fibroblasts, Spry4^S241^ expressing cells needed about 10 days to duplicate when seeded at a density of 10 to 20% confluency, while expression of Spry4^Y241^ caused a prolongation of the duplication time to about 15 days (Figure 4).

These data corroborate the conclusion that a mutation of Spry4 associated with Kallmann syndrome creates a more potent inhibitor of RTK-mediated processes in fibroblasts.

### 2.5. The Conversion of Serine 241 to a Tyrosine Enables Spry4 to Interfere with Cell Migration in an Osteosarcoma-Derived Cell Line

**Figure 5:**
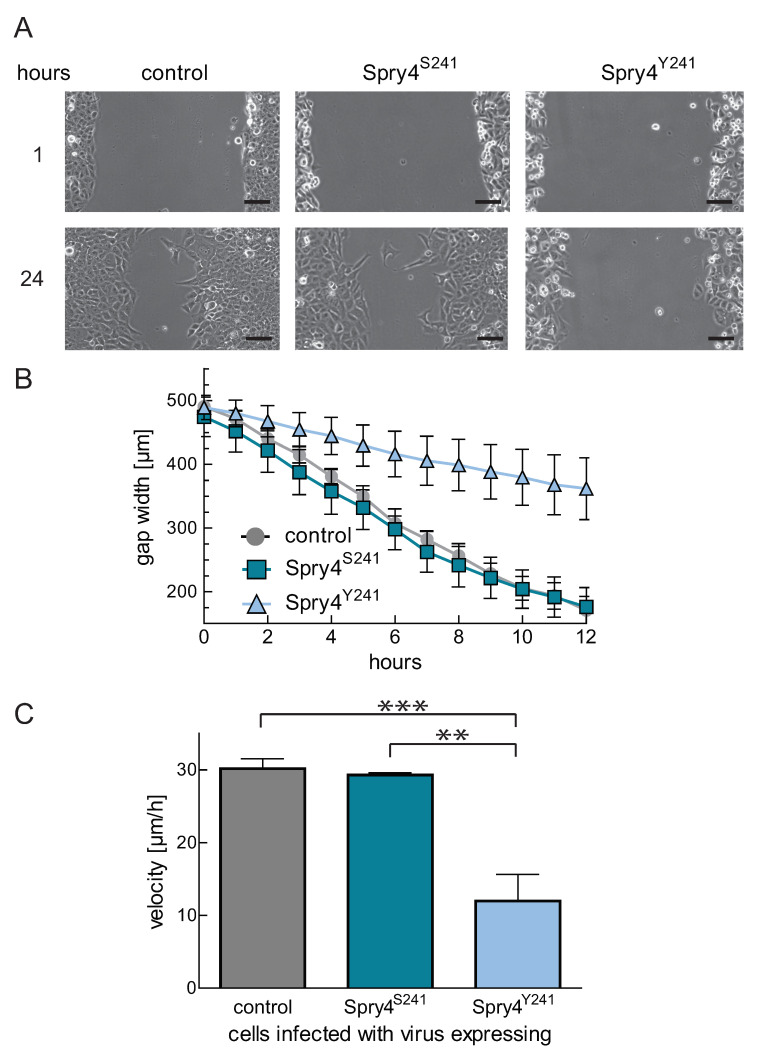
Influence of Spry4 expression on cell migration of osteosarcoma-derived cells. (**A**) Pictures of a representative scratch with 10× magnification at the indicated time points are shown. U2OS cells expressing the indicated proteins after viral infection were investigated by picturing the gap every hour using a VISITRON Live Cell Imaging System. Scale bar in the right corner of the pictures represents a distance of 100 μm. (**B**) A curve summarizing the distance coverage over a time period of 12 h by measuring gap widths of three replicative scratches in three experiments every two hours using ImageJ is depicted. (**C**) Applying a linear regression over the linear range of the curves, migration velocities of four experiments were calculated. Means of at the four experiments ± SEM are summarized as column bar. A one-way ANOVA test was used to acquire significance. ** *p* < 0.01; *** *p* < 0.001.

Next, we wanted to investigate the function of the Spry4 mutation associated with Kallmann syndrome in osteosarcoma-derived cells where the wildtype Spry4 protein is unable to interfere with cell migration [20]. To this end, U2OS cells were infected with viruses expressing either a control protein, wildtype Spry4^S241^, or Spry4^Y241^ and a Scratch assay was performed. When Spry4^Y241^ was expressed, the velocity of the cells invading the gap was strongly delayed as compared to control or wildtype Spry4 infected cells, in which the cell lawn was nearly closed after 24 h (Figure 5A). On average the cells expressing the mutated Spry4 need three times longer to close the gap and cover only a distance of about 12 µm per hour (compared to 30.19 ± 1.31 or 29.29 ± 0.33, respectively) (Figure 5B,C).

These data demonstrate that the alteration at position 241 can empower Spry4 protein to counteract the migratory potential of cancer cells originated from bone.

### 2.6. FGF-Induced Signaling in an Osteosarcoma-Derived Cell Line Is Primarily Inhibited by the Mutated Spry4 Protein

**Figure 6:**
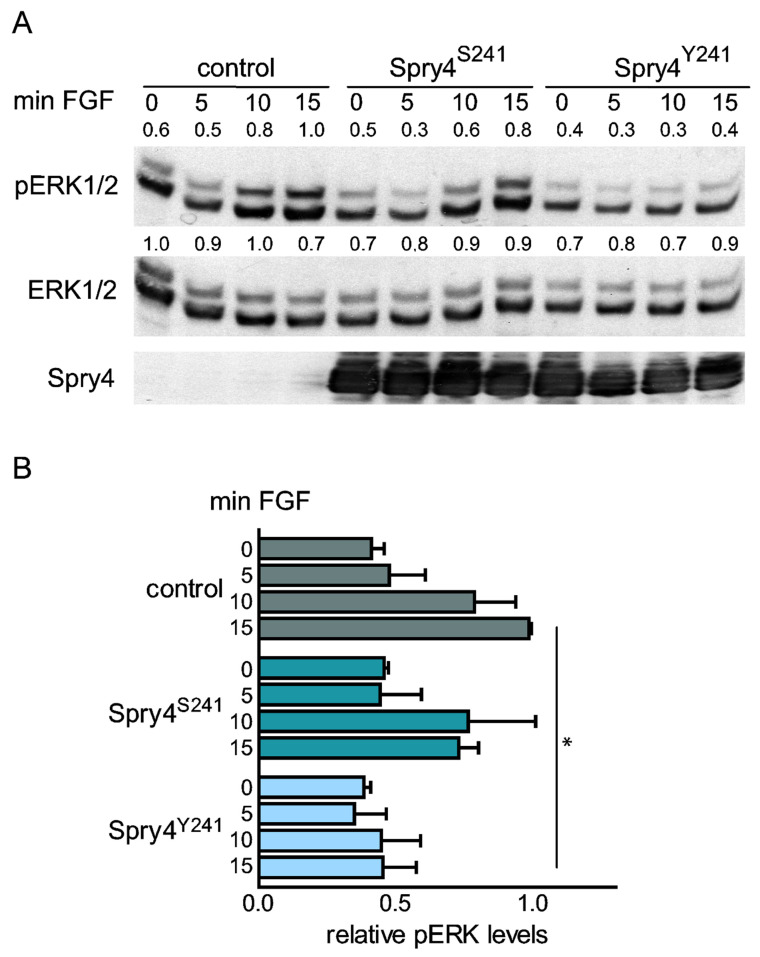
Influence of Spry4 proteins on FGF2-induced ERK activation in osteosarcoma-derived cell lines. U2OS cells were serum-deprived and next day infected with adenoviruses expressing luciferase (control), wildtype Spry4 (Spry4^S241^) or the mutated Spry4 protein (Spry4^Y241^). 72 h after serum removal, 10 ng/mL FGF2 were added for the indicated times. (**A**) Consecutively the immunoblots were treated with antibodies recognizing pERK1/2, total ERK 1/2 and Spry4. Representative Western blots are shown. pERK1/2 as well as ERK1/2 bands were quantified using ImageQuant 5.0 and the highest values were arbitrarily set as 1. (**B**) Values for pERK1/2 were normalized to the corresponding values obtained for ERK1/2 expression and the calculated mean values ± SEM are summarized. By using an unpaired *t*-test in GraphPad Prism, significance was determined. * *p* < 0.05.

To perform a cell signaling assay U2OS cells were serum-deprived and infected with viruses expressing the Spry4 variants or a control protein before signaling was induced by the addition of FGF. While in both, cells expressing control as well as unaltered Spry4 proteins, a considerable increase of phosphorylated ERK1/2 proteins could be observed after 10 min, the cells expressing the Spry4 mutant failed to respond to this stimulus (Figure 6A,B).

These results verify the finding that the modification of Spry4 at position 241 enhances the inhibitory potential of Spry4 in FGF-mediated signaling.

## 3. Discussion

Recently, a sequencing screen in individuals with congenital hypogonadotropic hypogonadism was applied to search for mutations within the FGF8 synexpression group. Within this screen several Kallmann syndrome-associated mutations including a C to A conversion at position 722 of the coding sequence in Spry4 were identified [5]. This mutation is a missense mutation creating a Spry4 protein variant harboring a tyrosine at position 241 of the amino acid sequence instead of a serine.

In the present study, we investigated the influence of this amino acid substitution by comparing the influence of this protein variant with its wildtype progenitor protein. Since the alteration is within the conserved C-terminus, in a domain crucial for the inhibition of the MAPK pathway, it was not surprising that the inhibitory impact of Spry4 was affected by the mutation. Furthermore, the conversion is in close proximity to the Raf1 binding site as determined by Sasaki et al. [8]. While in our studies Spry4 wildtype protein failed to influence the activity of MAPK in WI-38 cells as well as in osteosarcoma-derived cells in response to FGF2 induction, the conversion of the serine at position 241 empowered the protein to interfere with FGF-mediated signal transduction. Previous reports were able to show that ectopic expression of mSpry4 inhibits phosphorylation of overexpressed ERK2 [8,22] in 293 cells and Spry4 expression interfered with the FGF2 activation of ERK1/2 proteins in glioblastoma-derived cells [19], while Spry4 failed to influence FGF-induced ERK phosphorylation in pancreatic cells [23]. In response to FCS, Spry4 was inhibiting MAPK activation in breast cancer cells [18], while it was inefficient in osteosarcoma-derived cells [20]. These data indicate that the cellular background might determine the susceptibility of mitogen-induced ERK phosphorylation towards Spry4. Our data suggest that the amino acid at position 241 is critical for the restricted functionality of Spry4. In accordance with earlier reports [8,24,25], Spry4 failed to influence EGF-mediated MAPK activation and this function was not enabled by the alteration at position 241. In principle, these data support the multiple observations that in mammalians, Spry proteins are incapable to inhibit EGF-mediated signaling [26,27,28,29].

Furthermore, our results indicate that the alteration of serine 241 had no critical effect on Spry4 levels. The already available data suggest that Spry4 is not so vulnerable to regulated protein degradation induced by c-Cbl than the Spry2 family member [30,31,32], but other mechanisms contribute to the regulation of Spry4 levels by affecting its stability [33,34]. However, like it is the case for c-Cbl, the interaction sites of the ubiquitin ligases potentially able to affect stability of Spry4 are localized in the N-terminus of the protein [35,36].

Concerning the influence on cellular processes, we observed that the indicated weak interference potential of Spry4 wildtype protein with cell migration was augmented to a considerable slowdown of the cells in the presence of Spry4^Y241^. Corroborating, the tyrosine substitution capacitated Spry4 to interfere with proliferation of human embryonic lung fibroblasts. The inhibitory potential of Spry4^Y241^ on cell migration and proliferation is likely connected to the gained ability to interfere with MAPK. Several reports showing concordant inhibition of cell proliferation and migration like in breast [18] and brain [19] cancer-derived cells demonstrate that in the same cell lines Spry4 is able to interfere with this pathway. In cell systems where explicitly no effect of Spry4 on these biological processes was reported, like in osteosarcoma and ovary carcinoma [20,21], these incapacities were accompanied by an inefficient inhibition of MAPK. Nonetheless, in lung cancer-derived cells Spry4 is reported to interfere with proliferation as well as migration via the Wnt-pathway [17]. Additionally, several reports suggest that Spry4 can influence migration and proliferation independently. In cells derived from prostate and pancreatic carcinoma, Spry4 is inhibiting cell migration without affecting the proliferation of the cells [23,37]. Inhibition of cell migration by Spry4 is furthermore observed in endothelial cells [38]. Nonetheless, our data allow the conclusion that the substitution resulting from the Kallmann syndrome-associated Spry4 mutation creates a hyperactive form of Spry4. These data are in agreement with results showing that the Kallmann syndrome is caused by diminished signaling via the FGFR1 since, in contrast to the Craniosynostosis and chondrodysplasia syndromes [39] caused by point mutations resulting in excessive uncontrolled signaling, in Kallmann syndrome, the causal FGFR1 alterations are loss of function mutations [40]. This causes a reduced dosage of FGF mitogen action. The effect of the Spry4 alteration resulting in a hyperactivation of the protein towards FGF2-mediated signaling would lead to the same consequence: a reduction of FGF signaling.

In this way the mutation can contribute to the syndrome-associated phenotype. Additionally, our results can be important knowledge to increase the feasibility of Spry4 in targeted therapy of cancer.

## 4. Materials and Methods

### 4.1. Cell Lines

WI-38 cells are primary embryonic human lung fibroblasts which were purchased from the American Type Culture Collection (ATCC). All experiments were carried out with cells between passages 24 and 26. Medium was exchanged regularly every 2–3 days. The human osteosarcoma cell line U2OS were also purchased from the American Type Culture Collection. All cells were cultured at 37 °C in 7.5% CO_2_n Dulbecco’s modified Eagle medium (DMEM) containing 10% fetal calf serum (FCS) supplemented with penicillin (100 U/mL) and streptomycin (100 µg/mL).

### 4.2. Adenovirus Generation and Application

Adenoviruses expressing Spry4 and the control protein luciferase were already generated [20,41].

To generate adenoviruses expressing the Spry4^Y241^ protein, the cysteine at position 722 was exchanged by an adenine. Using two partially overlapping primers 5- GGCTACTGCGCTGACCACCCCTGC-3 (Spry4KS1-s) and 5- TCAGCGCAGTAGCCCTCATCGTCC-3 (Spry4KS1-as) and pADloxSpry4 as a template, site-directed mutagenesis was performed as described [42]. The correct introduction of the mutation into the Spry4 coding sequence was verified by sequencing analysis. Recombinant viruses were produced according to an earlier established protocol [43] and the concentration of the generated adenoviruses applied in the experiments was determined as described [19].

### 4.3. Cell Signaling Assay

For analyzing signal transduction pathways, cells were seeded into Ø6 cm tissue culture plates in medium containing 10% FCS in a density appropriate to achieve a 30% confluency 24 h later. Then, serum-withdrawal was initiated by washing the cells twice with serum-free medium. For starvation the cells were incubated for 3 days in DMEM containing no FCS but supplemented with penicillin (100 U/mL) and streptomycin (100 µg/mL). During the starvation time, at least 24 h after serum withdrawal cells were infected with adenoviruses. Signaling was then induced by the addition of 10 ng/mL EGF or 10 ng/mL FGF2, respectively. At well-defined time points after mitogen supplementation the cells were lysed by collecting them in sample loading buffer consisting of 10% glycerol, 5% ß-mercaptoethanol, 2.3% SDS, 62.5 mM TrisHCl (pH 6.8), and 0.001% bromphenolblue and subsequent boiling for 10 min.

### 4.4. Immunoblot

Immunoblotting was carried out as specified [44] using Spry4 antibodies raised to the N-terminal 60 aa of the protein, and purified as described [42]. Antibodies recognizing phosphorylated extracellular signal-regulated kinase (pERK) (#9101 were purchased from Cell Signaling Technology (Danvers, MA, USA) and diluted 1:1000. Furthermore, from Santa Cruz (Santa Cruz Biotechnology, Inc., Dallas, TX, USA) purchased antibodies directed against ERK 1/2 (sc-514302) and glyceraldehyde 3-phosphate dehydrogenase (GAPDH) (sc-365062) were used in a 1:500 and 1:20,000 dilution, respectively. The HRP-coupled secondary antibodies were purchased from GE Healthcare (Chalfont St. Giles, UK) and usually incubated in a 1:5000 dilution.

### 4.5. Scratch Assay

To perform a Scratch assay, 50% confluent cells were infected with adenoviruses expressing luciferase, wildtype Spry4 or Spry4^Y241^, respectively. Twenty-four hours post infection, cells were transferred into a 6-well plate. Next day, the serum was removed by washing the cells twice and following a two-day incubation period in serum free medium a Scratch assay was performed as earlier described [19]. For U2OS no serum deprivation was applied.

### 4.6. Growth Curve

Growth curves were performed using 10^5^ cells following an earlier described protocol [20]. Each experiment was repeated at least two times in duplicates.

## Data Availability

All data are included in the article.
